# Eurasian beaver ( *Castor fiber* ) mating behavior

**DOI:** 10.17912/micropub.biology.001454

**Published:** 2025-02-19

**Authors:** Henrik Bringmann

**Affiliations:** 1 Biotechnology Center, Center for Molecular and Cellular Bioengineering (CMCB), TU Dresden

## Abstract

Eurasian beavers ( *Castor fiber* ) typically mate in the water at night, and observations of mating beavers are extremely scarce. As a result, little is known about their mating behavior. Here, I provide video documentation of beavers mating in the River Elbe during daylight, offering a unique opportunity to observe their mating behavior. The mating cycle consisted of: 1) a phase of self-grooming on land, 2) followed by the male pushing the female into the water and following her, and 3) copulation taking place in the water. This cycle was repeated several times throughout the same day. These observations contribute to our limited understanding of Eurasian beaver mating behavior.

**Figure 1. Mating behavior of the Eurasian beaver:**
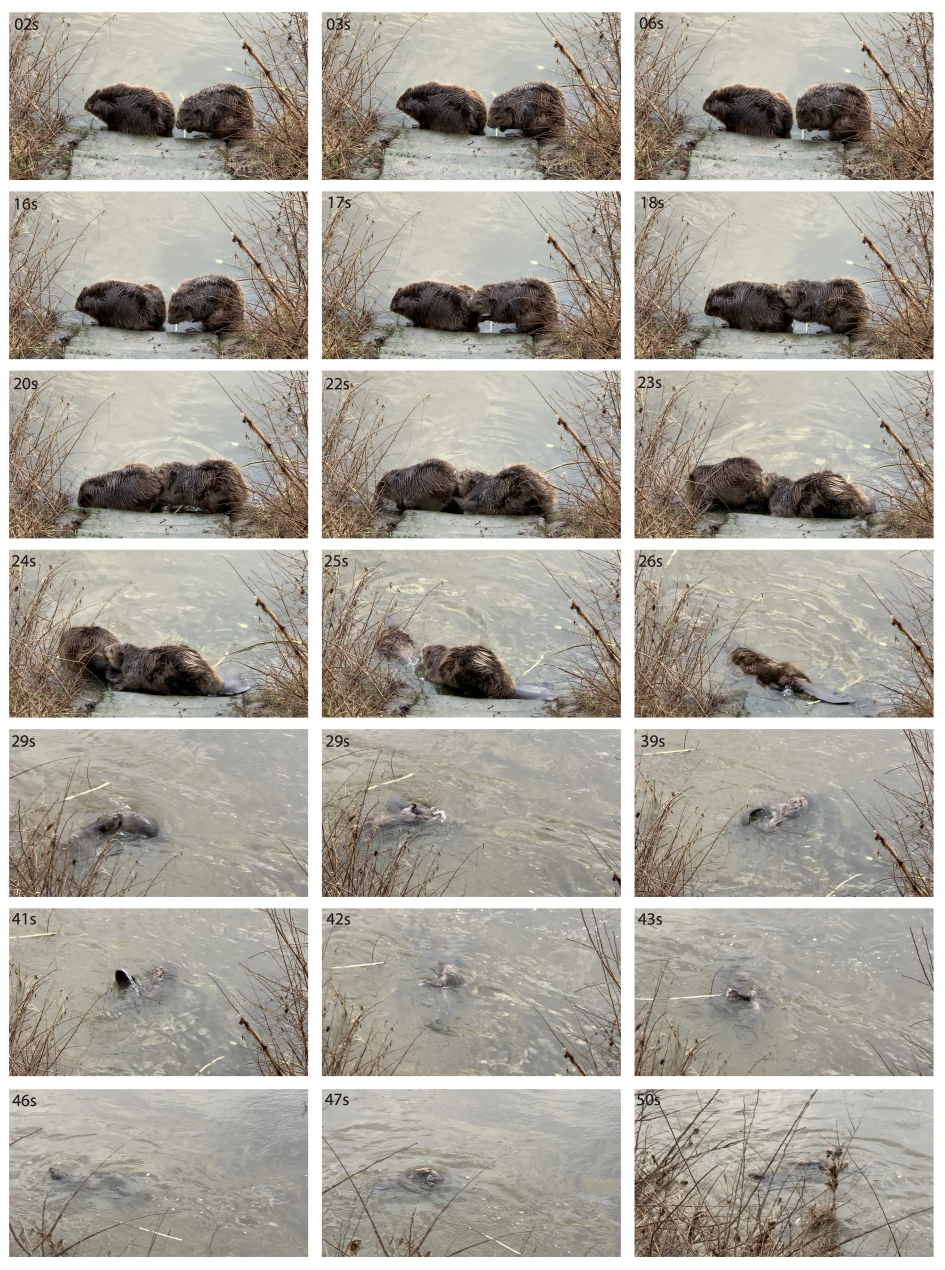
Frames extracted from Movie 2. The behavior is best observed in the associated movie files, Movies 1-2. From 0 to 16 seconds, the beavers are self-grooming. Between 17 and 25 seconds, the presumable male beaver pushes the other (presumably female) beaver into the water. At 26 seconds, the presumable male beaver follows the pushed beaver into the water. By 29 seconds, the pushing beaver grabs and holds onto the pushed beaver. Between 39 and 47 seconds, after a period underwater, the beavers appear at the surface and engage in copulation movements. At 50 seconds, the beavers appear at the surface and drift off with the current.

## Description

The mating behavior of Eurasian beavers in Germany was described in 1928 by A. Mertens, who had been observing beavers for nearly 40 years but had never personally witnessed mating, highlighting how rare such observations are. He summarized the accounts of three contemporaries: forester Backe, artist and animal painter E. Zehle, and forester Saleck. While Backe reported a potential case of mating on land, both Zehle and Saleck observed mating in water. Zehle had watched beavers mating at dawn or dusk in the Berlin Zoological Garden, while Saleck observed a beaver pair at Lake Goldberg (Loedderitz, Germany) in May 1927. Both reported that the beavers grasped one another in the water, pressing their ventral sides together. While lying slightly on their sides, the beavers engaged in pumping movements with their lower backs. Saleck noted a second round of mating 15 minutes after the first (Mertens, 1928).

In 2011, a 14-second video sequence of two beavers mating in Germany was reported to have been captured by an automatic surveillance camera, originally intended to document raccoons. This video confirmed that beavers mate in water and is likely the first video documentation of beaver mating in Germany ( https://www.youtube.com/watch?v=DkBjFU-REfY&t=14s ). The video appears to show actual copulation, but little is known about the behaviors associated with mating in Eurasian beavers. Observations of mating Eurasian beavers are extremely rare, and as a result, the full sequence of mating behavior in European beavers is poorly documented and does not seem to have been actively filmed in Germany prior to this.

I report here the observation and video recording of Eurasian beaver mating behavior on February 10th, 2024, in Laubegast (Dresden, Germany). Around 7 a.m., I spotted two beavers just a few meters away, engaging in a cycle of mating behavior consisting of: 1) intensive self-grooming while sitting on land near the water; 2) the presumptive male moving to the back of the presumptive female, pushing her into the water, and following her; 3) in the water, the two beavers held onto each other, potentially copulating; 4) afterward, the male swam behind the female until they exited the water and resumed self-grooming. Unfortunately, the first round of mating behavior was not captured on camera. A few minutes later, the cycle was repeated, and I was able to document it on video. During the presumptive copulation, the beavers were fully submerged, so it is unclear whether copulation actually occurred (Movie 1). The beavers then moved into the vegetation, making further observations difficult. Approximately 50 minutes later, the two beavers reappeared by a stone stair leading to the water and sat on the lowest step, just a few meters from the observer, which made subsequent observations easier. There, the mating cycle of 1) self-grooming, 2) the male pushing the female into the water, and 3) copulation in the water was repeated and documented on video again. This time, the thrusting movement of the lower back was clearly visible, indicating successful copulation ( Figure 1, M ovie 2).

In total, three cycles of mating behavior were observed. These observations support previous reports that Eurasian beavers copulate in the water and reveal a stereotyped cycle of moving in and out of the water. The cycle begins with self-grooming, followed by the male pushing the female into the water, and then copulation in the water. However, it is unclear from these videos whether copulation actually occurred or was only attempted. This method of copulation in water may be inherently inefficient, which could explain why multiple rounds of the cycle are carried out in a single day. Alternatively, it is possible that several rounds of successful copulation take place. Grooming was observed in all three cycles, suggesting that it is a typical part of the mating behavior, though additional observations are needed to confirm this. While the beavers groomed themselves, I did not observe one individual grooming the other.

Recognizing the sexes in beavers based on external observations is challenging. In this case, the beaver performing the 'pushing' behavior is interpreted as the male, though this interpretation requires further confirmation, and alternative explanations are possible.

While beavers are typically nocturnal and rarely visible during the day, these mating observations occurred at dawn, around 7-8 a.m., when the first daylight appeared. This shift in copulation activity to daylight hours might be speculatively influenced by the elevated water level of the Elbe River on that day.

This video documentation of Eurasian beaver mating behavior could contribute to a better understanding of this rarely observed behavior.

## Methods

Beavers were filmed with an iPhone X on 10 ^th^ February 2024.

During the preparation of this work, the author used chatgpt to review the text for clarity and correctness. After using this tool, the author reviewed and edited the content as needed and takes full responsibility for the content of the published article.

## Data Availability

Description: Movie 1. Resource Type: Audiovisual. DOI: https://doi.org/10.22002/y8j62-thh38 Description: Movie 1. Resource Type: Audiovisual. DOI: https://doi.org/10.22002/3s293-ps112
